# Antibacterial Activity and Mechanism of a Scorpion Venom Peptide Derivative *In Vitro* and *In Vivo*

**DOI:** 10.1371/journal.pone.0040135

**Published:** 2012-07-05

**Authors:** Luyang Cao, Chao Dai, Zhongjie Li, Zheng Fan, Yu Song, Yingliang Wu, Zhijian Cao, Wenxin Li

**Affiliations:** State Key Laboratory of Virology, College of Life Sciences, Wuhan University, Wuhan, P. R. China; Instituto Butantan, Brazil

## Abstract

BmKn2 is an antimicrobial peptide (AMP) characterized from the venom of scorpion *Mesobuthus martensii* Karsch by our group. In this study, Kn2-7 was derived from BmKn2 to improve the antibacterial activity and decrease hemolytic activity. Kn2-7 showed increased inhibitory activity against both Gram-positive bacteria and Gram-negative bacteria. Moreover, Kn2-7 exhibited higher antibacterial activity against clinical antibiotic-resistant strains such as methicillin-resistant *Staphylococcus aureus* (MRSA). In addition, the topical use of Kn2-7 effectively protected the skin of mice from infection in an *S. aureus* mouse skin infection model. Kn2-7 exerted its antibacterial activity via a bactericidal mechanism. Kn2-7 killed *S. aureus* and *E. coli* rapidly by binding to the lipoteichoic acid (LTA) in the *S. aureus* cell wall and the lipopolysaccharides (LPS) in the *E. coli* cell wall, respectively. Finally, the hemolytic activity of Kn2-7 was significantly decreased, compared to the wild-type peptide BmKn2. Taken together, the Kn2-7 peptide can be developed as a topical therapeutic agent for treating bacterial infections.

## Introduction

Drug resistance poses an increasing threat to global public health, and new antibiotic-resistant pathogens have continued to emerge [1], [2]. Methicillin-resistant *Staphylococcus aureus* (MRSA) is considered one of the most threatening pathogens due to the high mortality rate and increased medical costs associated with treating it. [3], [4]. New types of antimicrobial agents are urgently needed to respond to the threat of pathogens that evolve resistance against conventional antibiotics [5].

AMPs are distributed among a wide range of species, including insects, plants, humans, and even single-celled organisms [6]. The structures of AMPs generally present highly amphiphilic topologies, in which hydrophilic and hydrophobic side chains are located on opposite faces of the molecule [7], [8]. These peptides are potent antimicrobial agents against bacteria, fungi, viruses, and parasites, and several AMPs have been reported to inhibit the growth of MRSA [9], [10], [11]. Their broad spectrum activity and low potential to induce resistance make AMPs an attractive family of compounds with the potential to be developed as therapeutics agents. During the last decade, several antimicrobial peptides have been investigated as therapeutic agents [12], [13].

Scorpion venom contains a diversity of bioactive peptides that represent a tremendous resource for use in drug design and development [14], [15]. Moreover, several AMPs have been found in scorpion venom, including hadrurin [16], scorpine [17], opistoporins, parabutoporin [18], ISCTs [19], mucroporin [9] and StCT1 [20]. These scorpion venom peptides commonly exhibit cytolysis or microbial inhibition functions.

In a previous study by our group, we characterized an antimicrobial peptide, BmKn2, derived from the venom of the scorpion *Mesobuthus martensii* Karsch [21]. In the present study, a derivative of BmKn2, was designed to increase the antibacterial activity and reduce the hemolytic activity. Kn2-7 showed increased inhibitory activity against both Gram-positive bacteria and Gram-negative bacteria in contrast to BmKn2. Meanwhile, Kn2-7 could inhibit HIV-1 by direct interaction with viral particle [22]. Moreover, the hemolytic activity of Kn2-7 was significantly lower than that of BmKn2. In addition, Kn2-7 exhibited higher antibacterial activity against clinical antibiotic-resistant strains. Topical application of Kn2-7 effectively protected skin from infection in an *S. aureus* mouse skin infection model. Finally, the antibacterial mechanism of Kn2-7 was clarified in this study.

## Results

### Antimicrobial Screening of BmKn2 Derivatives

**Figure 1:**
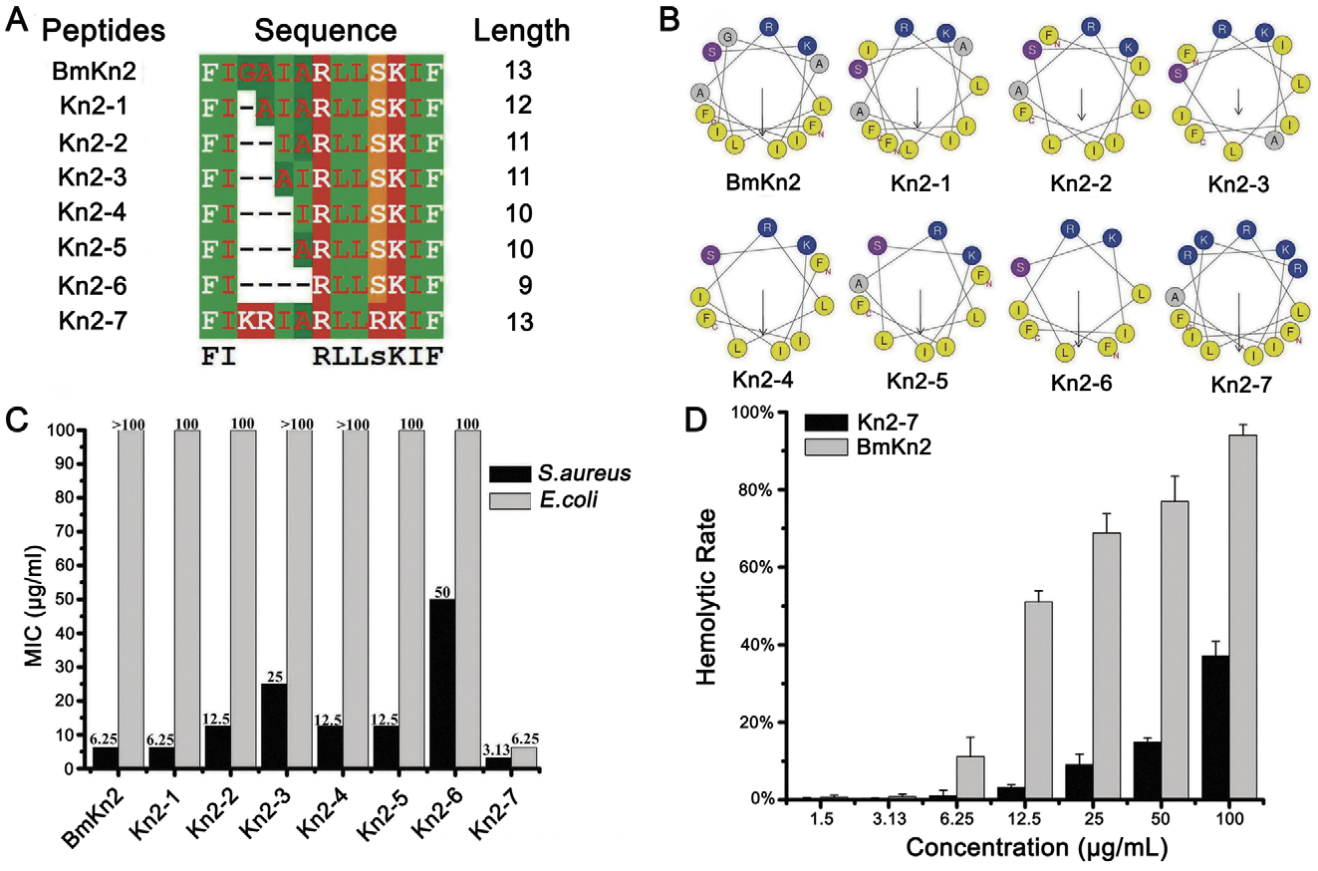
Screening, antimicrobial and hemolytic activities of Kn2-7 *in vitro*. (A) Multiple alignments of Kn2-7 and its seven derivatives. (B) Predicted secondary structure of Kn2-7 and its seven derivatives. (C) Antibacterial activity screening of Kn2-7 and its seven derivative peptides. (D) Hemolytic activity of Kn2-7. The hemolytic activities of the Kn2-7 and BmKn2 peptides were estimated by monitoring the increase in the absorbance at 570 nm after incubating human red blood cells with different peptide concentrations at 37 °C for 1 h. The positive control was 0.1% Triton X-100, and 0.85% saline was used as a blank.

Seven BmKn2-derived peptides were designed based on the amino acid sequence of BmKn2 (Figure 1A). The secondary structures of BmKn2 and its mutants were predicted to be 100% α-helical in nature. The helical wheels of BmKn2 and its mutants were divided into two parts, and the hydrophobic face and the hydrophilic face were labeled (Figure 1B).

These peptides were used to determine the inhibitory activities against the representative Gram-positive bacteria *S. aureus* AB94004 and the representative Gram-negative bacteria *E. coli* AB94012. The antimicrobial functional screening results showed that the Kn2-7 mutant exhibited the strongest inhibitory activity (Figure 1C).

The peptide Kn2-7 had little hemolytic activity at high concentration (Figure 1D). According to the method of Käber modified by Achmarine, the HC_50_ values of the mutant peptide Kn2-7 and the wild-type peptide BmKn2 were 90.27 µg/mL and 17.13 µg/mL, respectively (Figure 1D). Therefore, the hemolytic activity of Kn2-7 was significantly decreased, compared to the wild-type peptide BmKn2.

### *In Vitro* Antibacterial Activity of Kn2-7

In view of the *in vitro* antibacterial activity of Kn2-7, we selected it as the object of further study. Kn2-7 not only exhibited enhanced inhibitory activity against both Gram-positive bacteria and Gram-negative bacteria in contrast to wild-type BmKn2 (Table 1).

**Table 1:** MICs of Kn2-7 against Gram-positive and Gram-negative strains.

Strains	MIC (µg/mL)	
	Kn2-7	BmKn2
Gram-positive bacteria		
*S. aureus* AB94004	3.13	6.25
*S. aureus* ATCC 25923	3.13	6.25
*B. subtilis* AB91021	6.25	12.5
*B. thuringiensis* AB92037	6.25	12.5
*M. luteus* AB93113	6.25	6.25
Gram-negative bacteria		
*E. coli* AB94012	6.25	>100
*E. coli* ATCC 25922	25	>100
*P. aeruginosa* AB93066	25	50
*P. aeruginosa* A092994	50	>100
*P. aeruginosa* A093052	100	>100
*P. aeruginosa* A093056	50	>100
*P. aeruginosa* A093085	50	>100
*P. aeruginosa* A093115	100	>100

The antibiotic-resistant pathogens used in this study were clinical isolates, all of which were tested with corresponding antibiotics to verify their resistance prior to conducting experiments. As shown in Table 2, Kn2-7 was more effective against the antibiotic-resistant pathogens than the wild-type BmKn2.

**Table 2:** MICs of Kn2-7 against clinical isolated antibiotic-resistant bacterial strains.

Strains	MIC (µg/mL)				
	Kn2-7	BmKn2	Vancomycin	Penicillin	Cefotaxime
Penicillin resistant					
P1383	6.25	12.5	6.25	10000	6.25
P1389	6.25	12.5	6.25	10000	3.13
Methicillin resistant					
P1374	3.13	12.5	6.25	5000	400
P1369	3.13	12.5	6.25	20000	400
P1381	6.25	12.5	3.13	5000	400
P1386	6.25	12.5	3.13	10000	100
Penicillin sensitive					
P1111	3.13	6.25	3.13	25	6.25

All strains were clinical isolates obtained from the 302^nd^ Military Hospital, Beijing, China. All data are from experiments that were repeated at least three times with an MIC of no greater than 2 fold.

**Figure 2:**
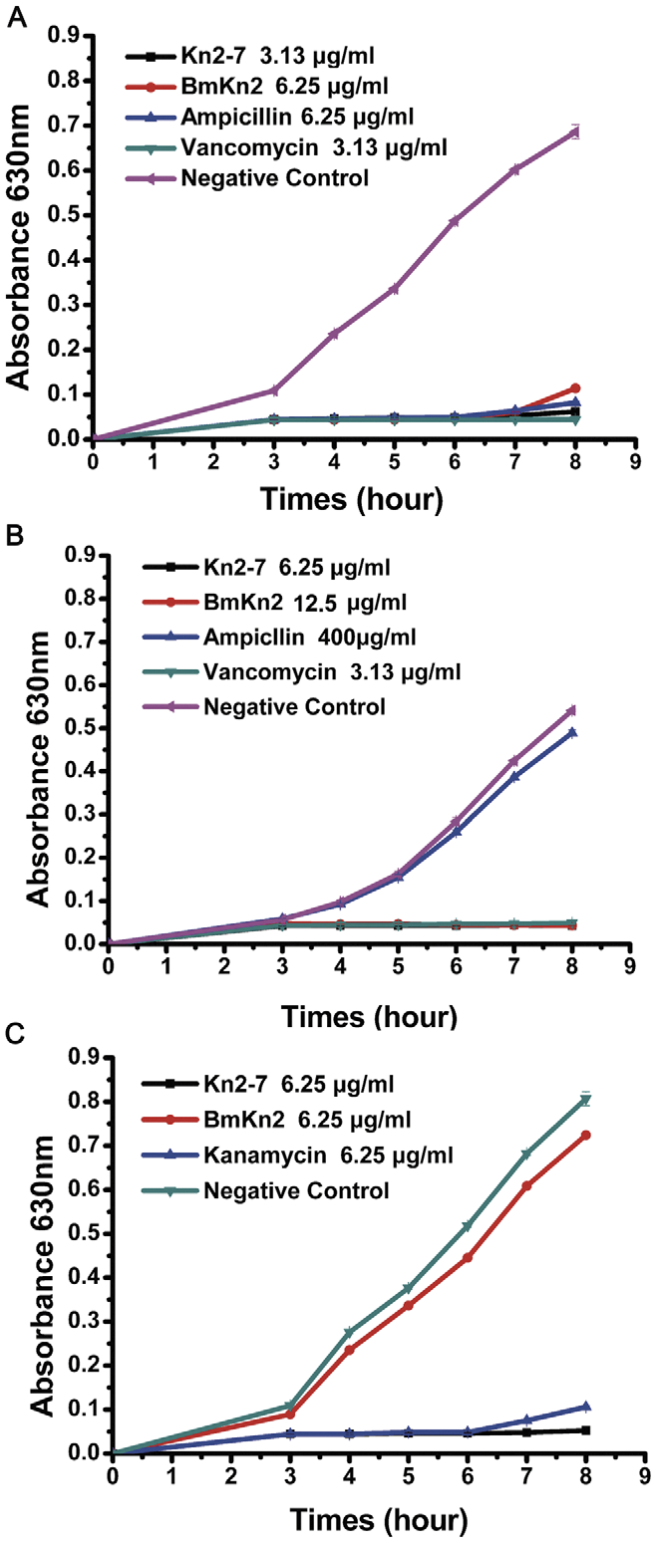
Gowth inhibitory activities of Kn2-7. (A) Growth curves of *S. aureus* AB94004 treated with Kn2-7, BmKn2 or antibiotics. (B) Growth curves of MRSA P1386 treated with Kn2-7, BmKn2 or antibiotics. (C) Growth curves of *E. coli* AB94012 treated with Kn2-7, BmKn2 or antibiotics.

Kn2-7 was not only able to inhibit the growth of standard strains, but it was also able to effectively inhibit the growth of clinically isolated antibiotic-resistant strains. As shown in Figure 2, at a concentration of MICs, Kn2-7 and BmKn2 both inhibited the growth of *S. aureus* completely. Additionally, Kn2-7 and BmKn2 inhibited the growth of MRSA P1386 completely. At a concentration of 6.25 µg/mL, the mutant peptide Kn2-7 and kanamycin both inhibited the growth of *E. coli* AB94012, while the wild-type BmKn2 peptide had no effect against *E. coli* AB94012.

### *In Vivo* Antibacterial Activity of Kn2-7

**Figure 3:**
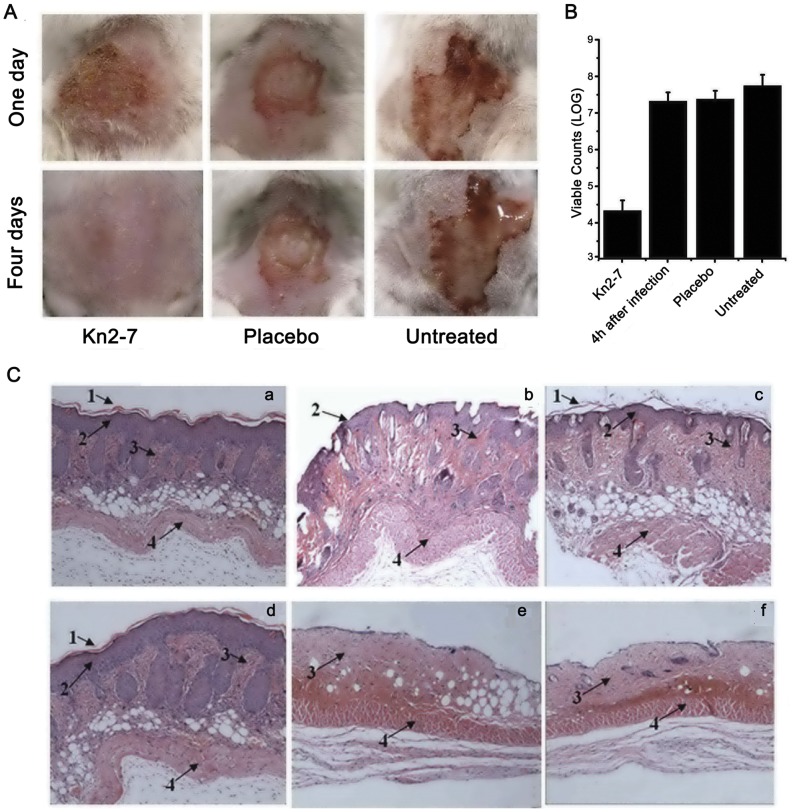
*In vivo* antibacterial activity of Kn2-7. (A) Mice were observed after being treated with the peptide on days 1 and 4. (B) Cutaneous viable counts in treated mice. Eight mice per group were euthanized, and the viable counts of the surviving *S. aureus* bacteria were then determined. (C) Histological morphologies of the skin in treated mice. (a) Normal dorsal skin of mice. (b) Immediately after the skin was scratched. (c) Four days after the skin was scratched. (d) Four days after *S. aureus* infection in skin treated with Kn2-7. (e) Four days after *S. aureus* infection in skin treated with BmKn2. (f) Four days after *S. aureus* infection in skin treated with a placebo. (g) Four days after *S. aureus* infection in untreated skin. Numbered arrows indicate the following: 1, corneum; 2, epidermis; 3, dermis; and 4, muscular layer.

To determine the *in vivo* antibacterial activity of Kn2-7, an *S. aureus* mouse skin infection model was established. After infection, the mice were observed daily. As shown in Figure 3A, one day after infection, the skin of the mice in the group treated with Kn2-7 had become scabbed over, while the wounded skin sections of the group treated with placebo were wet and presented with blistering. Additionally, blisters and light tissue fluid leakage could be observed in the wounded skin sections of the group that was not treated. Four days after infection, the skin sections of the group treated with Kn2-7 were healed, and their scabs were desquamated. However, the blisters in the skin sections in the group treated with placebo became larger. Furthermore, the skin sections exhibited a great amount of tissue fluid leakage in the group without treatment.

A group of mice was euthanized 4 hours after infection, and their skin viable counts were measured as a control. Other groups were euthanized 4 days after infection. As shown in Figure 3B, the colony counts in mice treated with the peptide Kn2-7 were clearly lower than those of the placebo-treated mice and the mice that were untreated.

The histological morphology of the skin sections was observed. As shown in Figure 3C, most of the epidermis of the scratched skin sections was defective, while other parts of the skin were normal compared to normal skin with intact structure. Four days after the skin of the mice was scratched, the epidermis recovered, and the biopsy specimens were observed to be normal. Four days after infection, the untreated group and the group treated with placebo lost nearly all of their epidermis, and the dermis was infected to a certain extent. However, the skin sections treated with Kn2-7 recovered almost fully and presented nearly intact structures.

### Antibacterial Mechanism

#### Secondary structure analysis

**Figure 4:**
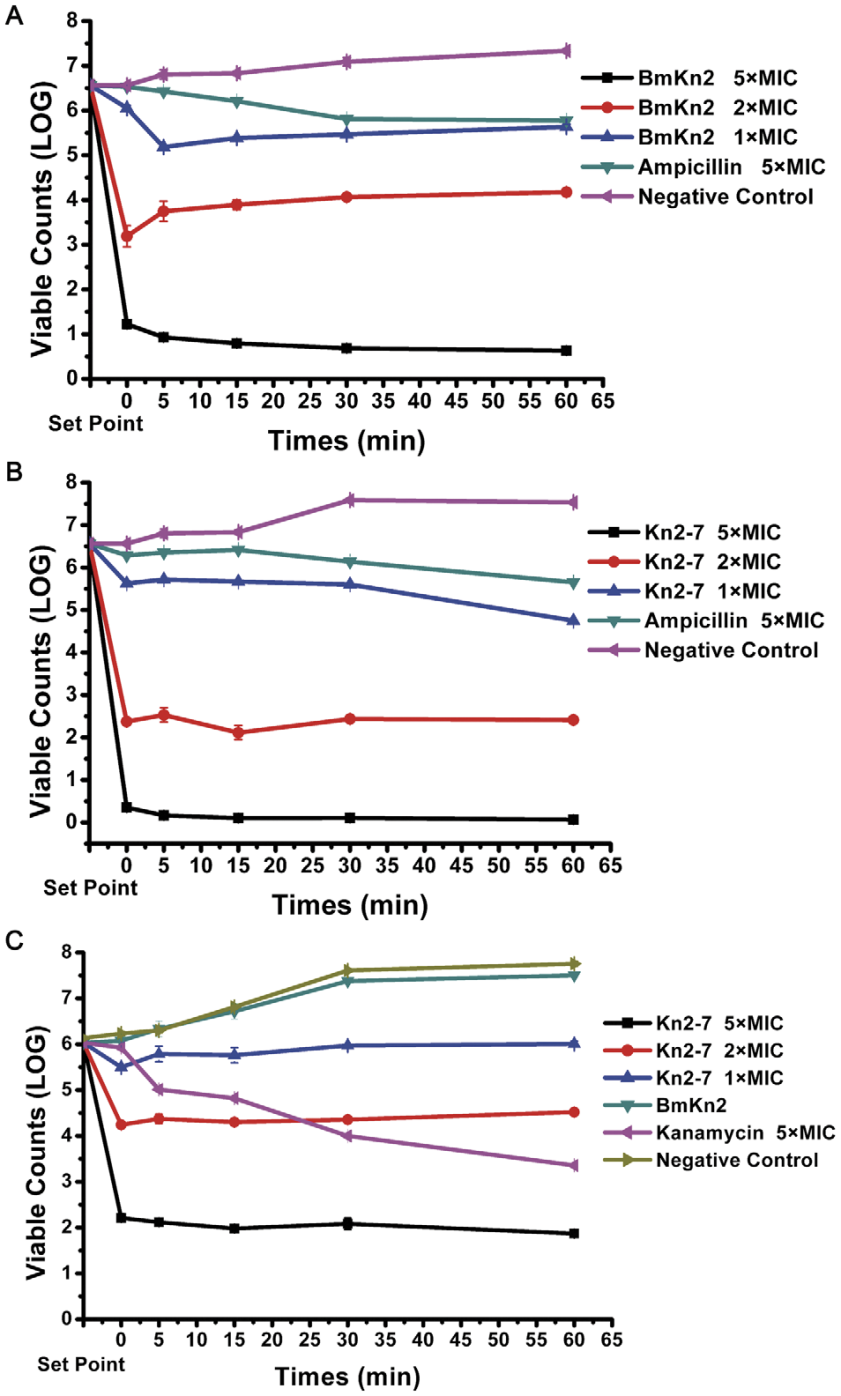
Secondary structure and enzyme release assay of Kn2-7. (A) and (B) Secondary structure analysis of the Kn2-7 and BmKn2 peptides. (A) Circular dichroism spectra of 0.1 mg/mL of Kn2-7 in water, 30% TFE/H_2_O or 70% TFE/H_2_O. (B) Circular dichroism spectra of 0.1 mg/mL of BmKn2 in water, 30% TFE/H_2_O or 70% TFE/H_2_O. (C) and (D) Enzyme release assay. (C) *S. aureus* AB94004 treated with Kn2-7 or BmKn2 was harvested, and the catalase activities of the supernatants were measured; 0.9% saline was used as the negative control, and ampicillin sodium was used as the antibiotic control. (D) *E. coli* AB94012 treated with Kn2-7 or BmKn2 was harvested, and the catalase activities in the supernatants were measured; 0.9% saline was used as the negative control, and kanamycin was used as the antibiotic control.

As shown in Figure 4A and Figure 4B, Kn2-7 and the wild-type peptide BmKn2 displayed typical α-helical profiles in the structure-promoting solution TFE (30% and 70%). However, both Kn2-7 and BmKn2 displayed random coil profiles in water.

#### Enzyme release assay of Kn2-7

The enzymatic activities of the supernatants were measured via an enzyme release assay immediately after treatment with Kn2-7 or BmKn2. As shown in Figure 4C, the supernatant from *S. aureus* AB94004 treated with ampicillin exhibited increasing catalase activity over time. However, the supernatant treated with Kn2-7 or BmKn2 showed almost the same catalase activity at each time point. As shown in Figure 4D, the supernatant from *E. coli* AB94012 treated with kanamycin presented increasing catalase activity over time. However, the supernatant treated with Kn2-7 showed nearly the same high enzymatic activity at each time point. In contrast, the supernatant treated with the wild-type peptide BmKn2 showed low catalase activity at each time point and did not exhibit increasing catalase activity over time.

#### Bactericidal effect of Kn2-7

**Figure 5:**
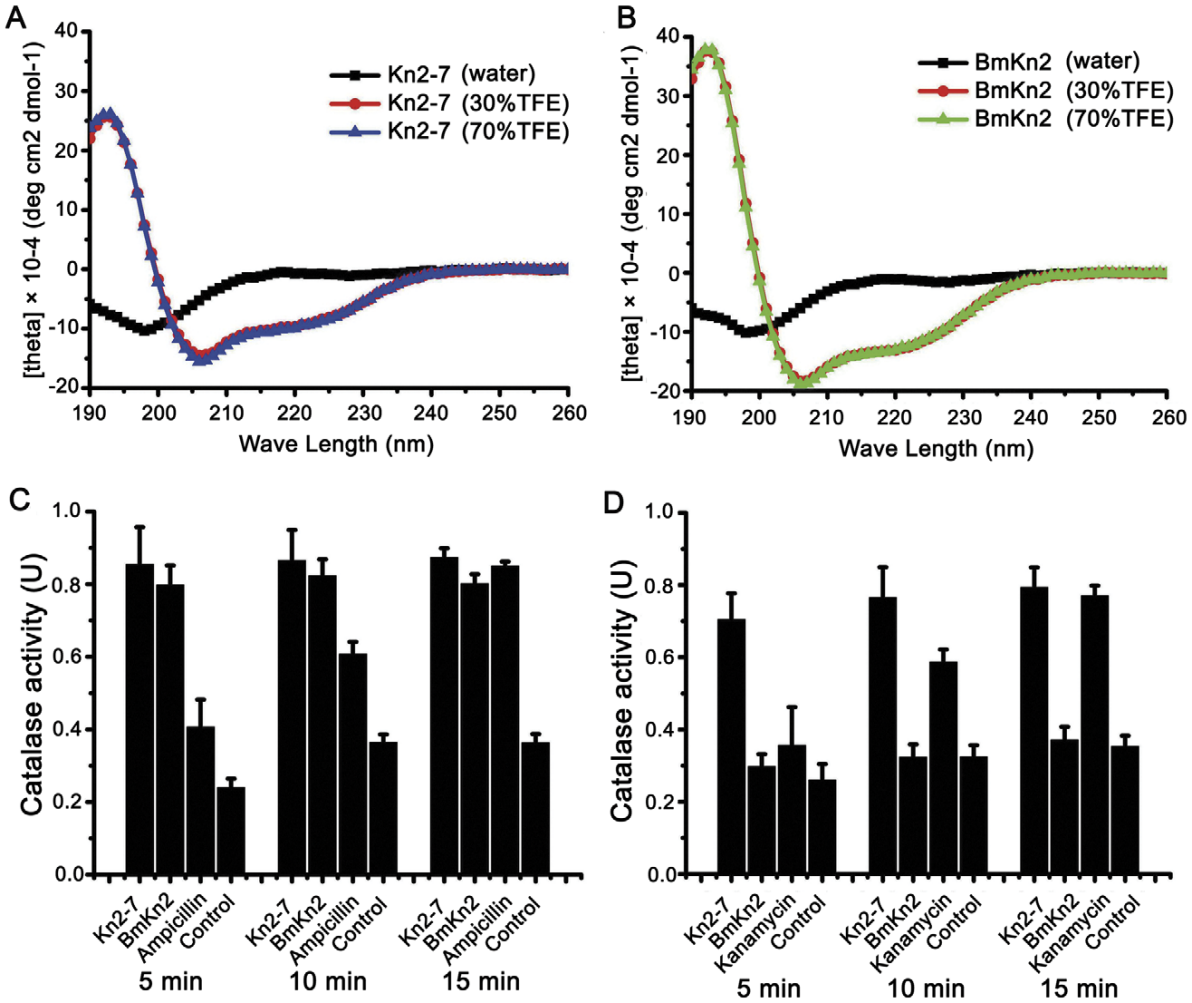
Bactericidal assays of the Kn2-7 and BmKn2 peptides against *S. aureus* and *E. coli* AB94012. Set point indicates untreated bacteria, and 0 minutes was defined as the time of the first sample collection, which was immediately after mixing bacteria and Kn2-7 or BmKn2. The other samples were collected at 5 min, 15 min, 30 min or 60 min. All of the counts were the average of three dishes, and the experiment was repeated at least three times. (A) Time-killing curve of Kn2-7 against *S. aureus* AB94004. (B) Time-killing curve of BmKn2 against *S. aureus* AB94004. (C) Time-killing curve of Kn2-7 against *E. coli* AB94012.

Kn2-7 exerted bactericidal effects on both *S. aureus* AB94004 and *E. coli* AB94012. As shown in Figure 5A and Figure 5B, when the peptide concentration increased, the killing rate also increased. Additionally, Kn2-7 was able to kill *E. coli* AB94012 rapidly, whereas the wild-type peptide BmKn2 was not able to kill *E. coli* AB94012 (Figure 5C).

#### Transmission electron microscopy

**Figure 6:**
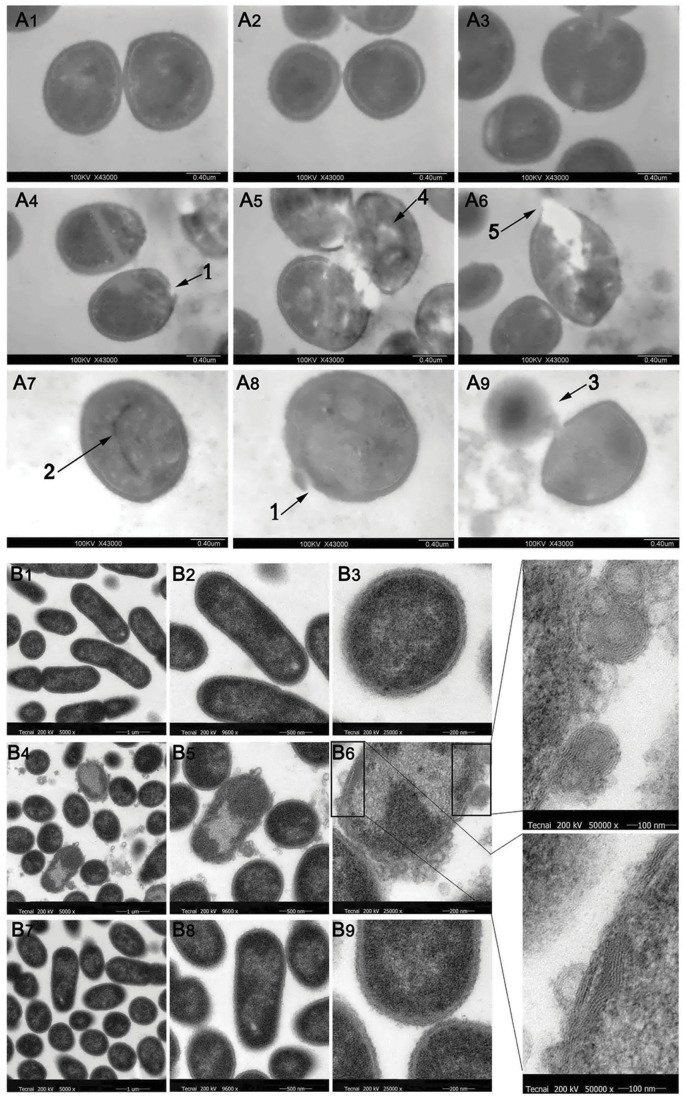
Transmission electron microscopy of *S. aureus* and *E. coli* treated with Kn2-7. (A) Transmission electron microscopy was performed with Kn2-7-treated *S. aureus*. (A1), (A2) and (A3) were negative controls. (A4), (A5) and (A6) show *S. aureus* treated with Kn2-7 for 2 min. (A7), (A8) and (A9) show *S. aureus* treated with BmKn2 for 2 min. (B) Transmission electron microscopy was performed with Kn2-7-treated *E. coli*. (B1), (B2) and (B3) were the negative controls. (B4), (B5) and (B6) show *E. coli* treated with Kn2-7 for 2 min. (B7), (B8) and (B9) show *E. coli* treated with BmKn2 for 2 min.

Semi-thin sections of *S. aureus* AB94004 and *E. coli* AB94012 treated with Kn2-7 or BmKn2 were prepared to observe the structural changes in the cell walls and membranes of the bacteria (Figure 6). Cracks were observed in the cell walls of the bacteria. Additionally, membrane disruption was observed in the peptide-treated bacteria. Furthermore, semi-thin sections of bacteria treated with Kn2-7 or BmKn2 showed that there was a release of cell content upon cell wall disruption. As shown in Figure 5B, BmKn2 had no effect against *E. coli* AB94012. However, Kn2-7 accumulated on the surface of *E. coli* AB94012, combined with the cell wall and membrane, and then formed microspheres that surrounded the bacteria.

#### Biolayer interferometry

**Figure 7:**
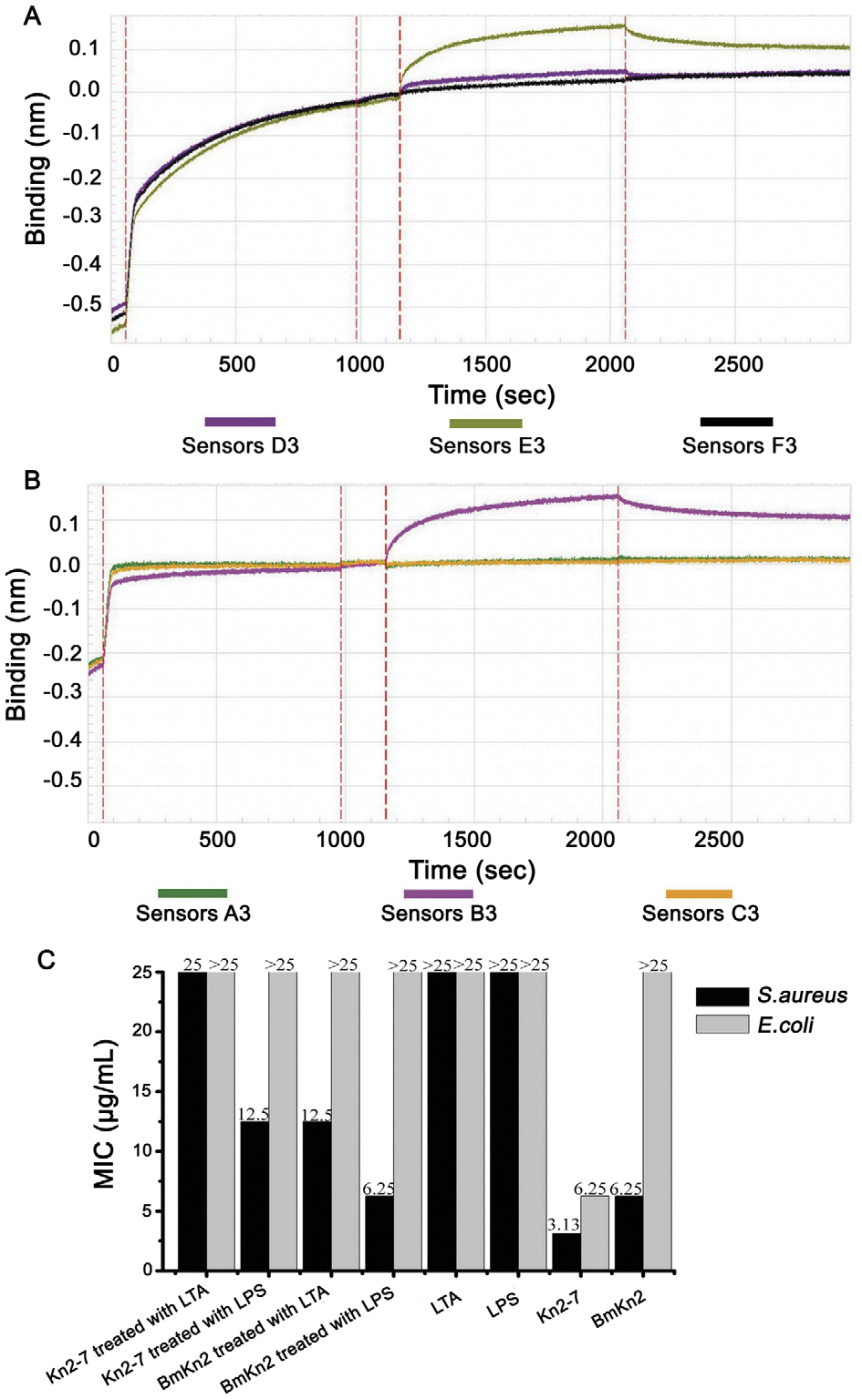
Binding assay of Kn2-7 and LTA or LPS. (A) Interaction of 10 µg/ml of Kn2-7 with 500 µg/ml of LPS or 500 µg/mL of LTA. Curve A3 is LPS, curve B3 is LTA and curve C3 is buffer. (B) Interaction of 10 µg/mL of BmKn2 with 500 µg/mL of LPS or 500 µg/mL of LTA. Curve D3 is LPS, curve E3 is LTA and curve F3 is the buffer. (C) MICs of Kn2-7 and BmKn2 treated with LTA or LPS against *S. aureus* and *E. coli.*

BLI was performed to identify the target of Kn2-7 in *S. aureus* and *E. coli*. LTA is the main component of the peptidoglycan layer surrounding Gram-positive bacteria, while LPS is the main component of the cell wall surrounding Gram-negative bacteria. As shown in Figure 7A and Figure 7B, curve B3 and curve E3 indicated that the designed peptide Kn2-7 bound strongly to LTA, as did the wild-type peptide BmKn2. Meanwhile, curve A3 and curve D3 indicated that only the Kn2-7 mutant was able to bind to LPS, while wild-type BmKn2 could not. LTA confer a portion of the negative charge associated with the peptidoglycans Gram-positive bacteria, and LPS confer a portion of the negative charge associated with the outer membrane surrounding Gram-negative bacteria, respectively.

#### LTA and LPS competition binding assay

To confirm the results of the BLI titrations, 250 µg/mL of Kn2-7 or BmKn2 was mixed with an equal volume of 1 mg/mL LTA or LPS. Then, the MICs of Kn2-7 or BmKn2 mixed with LTA against *S. aureus* and *E. coli* were measured. As shown in Figure 7C, the MICs of Kn2-7 and BmKn2 against *S. aureus* AB94004 were 3.13 and 6.25 µg/mL, respectively. The MIC of the Kn2-7 against *E. coli* AB94012 was 6.25 µg/mL. LTA and LPS exhibit no inhibitory activity against *S. aureus* or *E. coli*. The MICs of Kn2-7 and BmKn2 treated with LTA against *S. aureus* were 25 and 12.5 µg/mL, respectively. The MIC of Kn2-7 treated with LTA against *E. coli* AB94012 was at least 25 µg/mL. Meanwhile, the MICs of the Kn2-7 and BmKn2 treated with LPS against *S. aureus* were 12.5 and 6.25 µg/mL, respectively, and the MIC of the Kn2-7 mutant treated with LPS against *E. coli* was at least 25 µg/mL. All of these results further confirmed that both the Kn2-7 and BmKn2 peptides bind to LTA, but only the mutant Kn2-7 peptide was able to bind to LPS.

## Discussion

The structure-function relationship of some antimicrobial peptides has been analyzed to unravel the regular pattern of antibacterial activity, hemolytic activity and toxicity [23]. However, the regular patterns of antibacterial activity, hemolytic activity and toxicity have still need to be clarified. More recently, molecular design of antimicrobial peptides has become an important and attractive strategy for developing new antimicrobial drugs. In this study, six BmKn2-derived peptides were designed with the aim of increasing the proportion of polar residues at first. But the activity of the six deletion mutants did not improve. So Kn2-7 peptide was designed to increase the net positive charge of BmKn2 to improve its affinity for bacteria. The antibacterial activities of Kn2-7 were better than those of BmKn2. Moreover, Kn2-7 showed higher antibacterial activity than the other antimicrobial peptides identified from scorpion venoms to date [9], [11], [17], [18], [19], [20]. Furthermore, the hemolytic activity of Kn2-7 reduced observably compared to the wild-type peptide BmKn2. High hemolytic activity is always an obstacle to the application of AMPs. Increasing the net positive charge of AMPs may be a good way to improve the high hemolytic activity. Topical application of Kn2-7 cured the skin of mice infected with *S. aureus.* Compared with conventional antibiotics, Kn2-7 has many advantages, such as broad-spectrum activities, rapid killing ability, low levels of induced resistance, and broad anti-inflammatory activities. Kn2-7 may have the therapeutic potential for topical use.

New AMPs have been continuously isolated and identified. Several bilayer interaction and disruption models have been proposed for those AMPs that depend on membrane interference for their antibacterial activity, such as “barrel-stave pore”, “carpet mechanism”, “toroidal pore” and “disordered toroidal pore” [24]. New molecular models has been proposed to describe the mechanism more accurately, such as “form ion channels” [25] and “internalization into the cytoplasm” [26]. In the present study, the results of secondary structure analysis, bactericidal assays, enzyme release assays provided solid evidence that the inhibitory effect of Kn2-7 on the bacteria was mediated through rapid killing. However, the binding mode of Kn2-7 to the surface of *E. coli* seems different from that of *S. aureus.* The results of biolayer interferometry and competition binding assay proved to be so. The findings of TEM indicated that the bacterial cell wall of *S. aureus* was disrupted immediately upon treatment with Kn2-7. Kn2-7 accumulated on the surface of *E. coli*, combined with the outer membrane, and then formed microspheres surrounded the bacteria. This mode is consistent with the model of carpet mechanism in general. To the best of our knowledge, the structures of the microspheres were first observed in the antibacterial mechanism study and our results provide conclusive evidence for the model of carpet mechanism.

AMPs have both a cationic and amphiphilic nature. Their positive net charge ensures that AMPs can accumulate at the surface of bacteria that contain anionic polymers, such as LTA and LPS. Then, these peptides interact with the membranes of the microbes, resulting in membrane disruption [27]. The results of BLI titrations and competition binding assays showed that Kn2-7 disrupted the cell walls of *S. aureus* and *E. coli* through binding to LTA and LPS, respectively. The question of why the mutant peptide Kn2-7 can bind to both LTA and LPS while the wild-type peptide BmKn2 can bind to only LTA is of particular interest. Kn2-7 has five net positive charges while BmKn2 only has two. Therefore we speculate that increasing the numbers of positively charged residues turned out to have improved the affinity to the bacterial surface.

In conclusion, we described both *in vitro* and *in vivo* antimicrobial activities of Kn2-7, a scorpion venom peptide derivative, against Gram-positive bacteria and Gram-negative bacteria including clinical isolates, MRSA, MRCNS, PRSA and PRSE. The antimicrobial and hemolytic activities of Kn2-7 peptide were notably improved compared to the wild-type peptide BmKn2. The binding mode of Kn2-7 to the surface of *E. coli* was different from that of *S. aureus*. The peptide Kn2-7 disrupted the cell surface structure of *S. aureus* and *E. coli* via binding to LTA and LPS, respectively. Taken together, these data strongly suggest that Kn2-7 peptide can be developed as a topical therapeutic agent for treating bacterial infections.

## Materials and Methods

### Strains

*S. aureus* AB94004, *S. aureus* ATCC 25923, *E. coli* AB94012, *E. coli* ATCC 25922, *Pseudomonas aeruginosa* AB93066, *Bacillus thuringiensis* AB92037, *Bacillus subtilis* AB91021, and *Micrococcus luteus* AB93113 were purchased from the China Center of Type Culture Collection (CCTCC).

Clinical isolated *Pseudomonas aeruginosa* strains A092994, A093052, A093056, A093085 and A093115 were obtained from Hubei Maternal and Child Health Hospital.

Clinical isolates were obtained from the 302^nd^ military hospital of Beijing, China, including penicillin-resistant *S. aureus* (PRSA) P1383, penicillin-resistant *S. epidermidis* (PRSE) P1389, methicillin-resistant *S. aureus* (MRSA) P1381, P1386 and P1374, methicillin-resistant coagulase-negative *Staphylococcus* (MRCNS) P1369, and penicillin-sensitive *S. epidermidis* (PSSE) P1111.

### BmKn2 Derivatives and Chemical Synthesis

Six deleted mutants and one point mutant were designed with the aim of enhancing the net positive charge of the hydrophilic side of the peptide while maintaining the amphipathic character of the peptides. BmKn2 and the seven derivatives of this peptide noted above were synthesized with amidated C-termini by More Biotech (Wuhan) Ltd., China.

### Hemolytic Activity

The hemolytic activities of Kn2-7 and BmKn2 peptides were tested against human red blood cells [9]. HC_50_ values were obtained according to the method of Käber, modified by Achmarine [28]. Saline solution at a concentration of 0.85% was used as the negative control, and 0.1% Triton X-100 was used as the positive control.

### Antimicrobial Assays *in Vitro*

#### Minimum inhibitory concentration (MIC) analysis

The MIC was determined via a microdilution assay in 96-well microtiter plates according to the broth microdilution guideline of Clinical and Laboratory Standards Institute (CLSI) [29].

#### Growth inhibitory assay

The time growth curves of *S. aureus* and MRSA treated with BmKn2 or its derivatives were determined based on measurements taken at 630 nm at each time point. Different concentrations of peptide were added to the tested strains, which were cultured in 96-well plates using the same method as was used for determining the MIC.

### Antimicrobial assays *in vivo*

#### Experimental animals

Female BALB/c mice weighing 18–20 g were purchased from the Hubei Research Center of Laboratory Animals. All animal studies were approved by the Institutional Animal Care and Use Committee of Wuhan University.

#### Gel preparation

Kn2-7 or BmKn2 was prepared in gel form in 0.5% hydroxypropylcellulose to a final concentration of 5 mg/mL.

#### Mouse skin abrasion and infection model

Mice were injected with 150 mg/kg of cyclophosphamide 4 days prior to infection. Subsequently, the mice were injected with 100 mg/kg of cyclophosphamide 1 day prior to infection. After being anesthetized, the dorsal skin of the mice was shaved, and abrasions were produced in a 1×1 cm^2^ area using a blade. These abrasion wounds damaged only the stratum corneum and upper layer of the epidermis, but not the dermis. Five minutes later, the wounds were inoculated with 20 µl of *S. aureus* (10^7^–10^8^ CFUs) using a micropipettor. A group of mice was killed 4 h after infection to control for the infectious dose. Four hours after infection, the wounds of the other mice were smeared with 20 µl of 5 mg/mL gel or a placebo gel, which was used as a negative control. The treatment lasted for 4 days, with two peptide gel applications being performed daily (morning and evening at 8 h intervals). After 4 days of treatment, the mice were killed, and the wound area of the skin was immediately excised and homogenized. Suitable dilutions of the homogenates were plated on LB plates to determine the number of living bacteria (CFUs).

#### Histological examination

After the animals were euthanized, biopsy specimens of the excised wound area of the skin were collected and immediately fixed in phosphate-buffered formalin (10%, pH = 7.4). Then, the biopsy specimens were embedded in paraffin and stained with hematoxylin and eosin.

#### Statistical analysis

In each experiment, the mean CFU was calculated using log_10_-transformed data. Based on the averages of three experiments, the mean, range, and coefficient of variation were calculated.

### Antibacterial Mechanism

#### Secondary structure analysis

The secondary structures of Kn2-7 and BmKn2 were analyzed using Heliquest (http://heliquest.ipmc.cnrs.fr/cgi-bin/ComputParams.py) and circular dichroism (CD) assays. The CD assay was performed at room temperature in nitrogen-flushed cells using a 2-mm path with a Jasco J-810 spectropolarimeter. Kn2-7 and BmKn2 were measured at concentrations of 0.1 mg/mL in water, 30% TFE/H_2_O or 70% TFE/H_2_O. The peptide configuration was obtained by analyzing the data with CD deconvolution software.

#### Enzyme release assay

Bacterial suspensions and serial-diluted peptides were mixed at a ratio of 4∶1 in different tubes with a final volume of 2 ml. The mixtures were then incubated at 37°C with continuous shaking. At each time point, 200 µl of the treated bacterial suspension was transferred to sterilized tubes. After centrifugation, the supernatants were transferred to a sterilized 1.5-ml tube, and catalase activity was measured with a Catalase Assay Kit (Beyotime, Jiangsu, China) using the method for measuring peroxidase activity.

#### Bactericidal assay

Bactericidal curves for Kn2-7 and BmKn2 were determined against *S. aureus* AB94004 and *E. coli* AB94012. A 400 µl aliquot of the peptide solution was added to 1,600 µl of a bacterial suspension. At each time point, 200 µl of the treated bacterial suspension was transferred to a sterilized 1.5-ml tube. After centrifugation at 1,000×*g* for 5 min, the supernatant was removed, and the pellet was resuspended in 200 µl of LB medium. The bacterial suspensions were placed on agar plates and incubated at 37°C until viable colonies could be counted.

#### Transmission electron microscopy

Overnight-cultured S. aureus AB94004 and E. coli AB94012 were transferred to LB medium and cultured to the exponential phase. A 200 µl aliquot of the peptide solution was added to 800 µl of the bacterial suspensions at the final concentration of 1×MIC. The samples were cut into semi-thin sections. Microscopy was performed using a HITACHI H-8100 Transmission Electron Microscope.

#### Biolayer Interferometry

Biolayer Interferometry (BLI) experiments were performed on OCT RED (FortéBio, Inc.). A total of 500 µg/mL of lipoteichoic acid from *S. aureus* (LTA, Sigma, L2515), lipopolysaccharides from *E. coli* (LPS, Sigma, L2880) or buffer (10 mM HEPES, pH 7.4 and 0.14 M NaCl) was dispensed into 96-well microtiter plates at a volume of 200 µl per well. The operating temperature was 30°C. Biosensor tips (FortéBio, Inc., Menlo Park, CA) were pre-wetted with the buffer for 60 seconds at baseline. Then, 10 µg/mL Biotin-Kn2-7 or Biotin-BmKn2 was loaded onto the biosensor tips for 900 seconds. Baseline 2 was subsequently established for 300 seconds. Dissociation of Kn2-7 or BmKn2 occurred for 900 seconds in the buffer. Data were generated automatically by the Octet® User Software (version 3.1) and were subsequently analyzed from the resultant text files using Excel 2000.

#### LTA and LPS competition binding assay

A total of 250 µg/mL of each peptide solution was mixed with an equal volume of 1 mg/mL of the LTA or LPS solution. Then, the MICs of BmKn2 and Kn2-7 treated with LTA or LPS against *S. aureus* AB94004 and *E. coli* AB94012 were measured.
