# Correction: Antibacterial Activity and Mechanism of a Scorpion Venom Peptide Derivative *In Vitro* and *In Vivo*

**DOI:** 10.1371/journal.pone.0315211

**Published:** 2024-12-04

**Authors:** Luyang Cao, Chao Dai, Zhongjie Li, Zheng Fan, Yu Song, Yingliang Wu, Zhijian Cao, Wenxin Li

There is an error in Fig 3. Panel ’g’ is missing from Fig 3. Please see the correct Fig 3 here.

**Fig 3:**
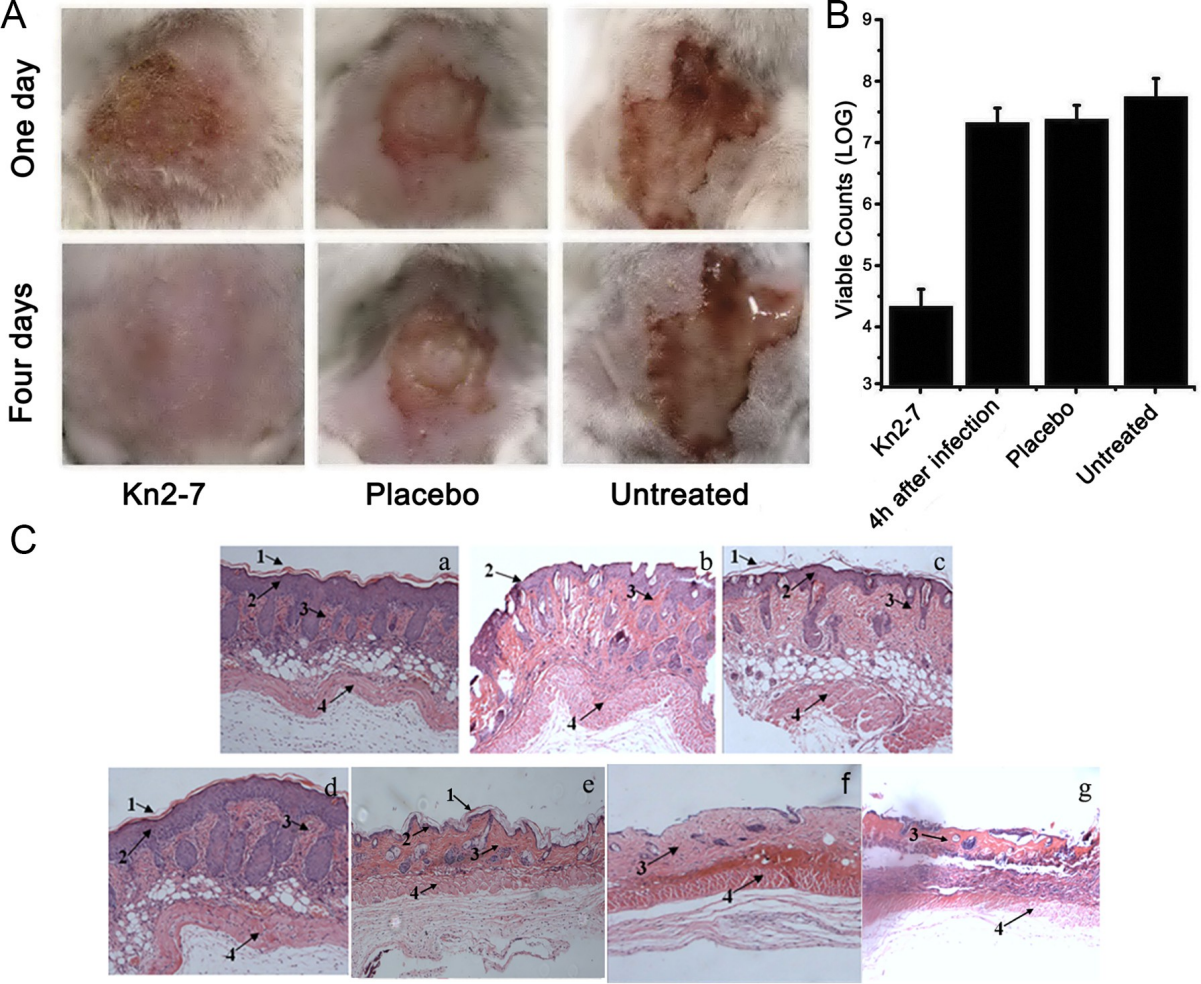
*In vivo* antibacterial activity of Kn2-7. (A) Mice were observed after being treated with the peptide on days 1 and 4. (B) Cutaneous viable counts in treated mice. Eight mice per group were euthanized, and the viable counts of the surviving *S*. *aureus* bacteria were then determined. (C) Histological morphologies of the skin in treated mice. (a) Normal dorsal skin of mice. (b) Immediately after the skin was scratched. (c) Four days after the skin was scratched. (d) Four days after *S*. *aureus* infection in skin treated with Kn2-7. (e) Four days after *S*. *aureus* infection in skin treated with BmKn2. (f) Four days after *S*. *aureus* infection in skin treated with a placebo. (g) Four days after *S*. *aureus* infection in untreated skin. Numbered arrows indicate the following: 1, corneum; 2, epidermis; 3, dermis; and 4, muscular layer.
